# Characteristics and Management of Emergency Department Patients Presenting with C2 Cervical Spine Fractures

**DOI:** 10.1155/2019/4301051

**Published:** 2019-05-15

**Authors:** Allison Tadros, Melinda Sharon, Kristen Craig, William Krantz

**Affiliations:** ^1^Department of Emergency Medicine, West Virginia University School of Medicine, 1 Medical Center Dr., Morgantown, WV 26506, USA; ^2^Department of Radiology, West Virginia University School of Medicine, Morgantown, WV, USA

## Abstract

**Background:**

C2 cervical fractures account for approximately 18% of cervical spine injuries. Few studies have examined patients presenting to an emergency department (ED) with this injury relative to demographics, injury mechanism, and hospital course.

**Objectives:**

To compare multiple variables of ED patients presenting with these types of injuries.

**Methods:**

In this retrospective cohort study, data were obtained from the Trauma Registry of an academic trauma referral center from January 1, 2011, to December 31, 2015. Patients who presented with a C2 fracture were identified. Information regarding the patient's gender, age, mechanism of injury, associated injuries, if a procedure was required, disposition, and mortality was extracted. Comparative analyses were conducted between cases over or under age 60.

**Results:**

Between January 1, 2011, to December 31, 2015, a total of 139 patients with C2 fractures were identified. Most patients were 60 years or older (79%). Of those, 62% were female, and falls were the most common mechanism (78%). Of those under 60, 50% were female and motor vehicle crashes (MVCs) were the most common mechanism (71%). Odontoid fractures comprised 84% of C2 fractures. Only 6% had an associated spinal cord injury. Less than one-third of patients required operative intervention for their spinal injuries, and intervention was more common in older patients. Following admission, 19% of patients required placement into a nursing home or skilled nursing facility.

**Conclusions:**

C2 fractures are more common in older adults and usually resulted from falls. Odontoid fractures are most common. Most C2 fractures do not result in neurologic injury, and only a third were treated surgically. However, several patients were unable to return to their homes following their injury.

## 1. Introduction

An estimated 153,461 patients suffered from a C2 cervical spine fracture in the United States (U.S) from 2000 to 2010 [1]. C2 fractures account for approximately 18% of all cervical spine injuries, with an increasing incidence of these fractures over the past decade [1–3]. The incidence of C2 fractures has been rising particularly for older adults, with one study describing a tripling of the rate in this population over a 14-year period [4]. In 2010, the estimated overall cost for treating C2 fractures in the United States was greater than $1.5 million [1, 3].

Prior literature has shown that C2 cervical spine fractures are common following both high-energy trauma such as MVCs and low-energy trauma like falls [4]. Younger patients are more likely to sustain a C2 fracture following a high-energy trauma, whereas low-energy trauma is the most often cited mechanism of injury for older patients [2, 5, 6]. Of those older patients, over half sustained a C2 fracture due to a ground level fall compared to younger populations, in which about 80% of these injuries are due to MVCs [2]. Mortality rates for patients with a cervical spine fracture without associated spinal cord injury were higher among those over 65 years of age (11.8%) versus those under 65 years of age (0.7%) and rates increase as patients age [5, 7].

There are several types of C2 fractures. There are 3 different classifications of odontoid fractures based upon the location of the fracture. Type I odontoid fractures involve the tip of the odontoid process, type II fractures go through the base of the of the odontoid process, while a type III fracture extends into the body of C2 [8]. A hangman's fracture is a fracture involving both of the pars interarticularis of C2, resulting in a subluxation of C2 on C3 [9]. Fractures can also involve the vertebral body of C2 or multiple fracture types (complex).

With an increasing incidence of C2 cervical fractures, it is useful to examine how age, associated injuries, and mechanism may affect their outcome. The purpose of the present study was to compare multiple variables of ED patients presenting with these types of injuries, including age, gender, mechanism, management, associated injuries, mortality, ED disposition, and hospital disposition.

## 2. Materials and Methods

In this retrospective cohort study, data were obtained from our institution's Trauma Registry from January 1, 2011, to December 31, 2015. The Trauma Registry collects data on all patients that require a trauma team activation in our academic Level 1 Trauma Center, which is a regional referral center for both pediatric and adult trauma patients. There were an average of 3538 patients entered annually over the 5 years of the study. There are 6 Trauma Registrars that are dedicated to the Trauma Program. All of the Registrars have degrees in Health Information Management and undergo at least one year of orientation to the Trauma Registry application (Collector), data abstraction, and report writing. Data is abstracted according to the National Trauma Data Bank data rules, Trauma Quality Improvement Program process measures. Data is abstracted from the electronic medical record and entered into Collector Trauma Registry database. Data is run from Collector using the application report writer. Data is entered on a daily basis from patient identification for the entire admission to discharge, both concurrently and retrospectively.

Patients who were diagnosed with a documented C2 fracture by computed tomography (CT) or magnetic resonance imaging (MRI) were identified; information regarding patients' age, gender, mechanism of injury, associated injuries, spinal intervention, disposition, and mortality were also extracted from the registry. The Trauma Registry was unable to discern past a high level cervical spine fracture (C1-C4), thus dictating the necessity of a chart review of the radiology report to abstract those cases which were specifically C2 injuries. To be included in the registry, the cervical spine diagnosis could have been made at a transferring facility or at any time during hospitalization at our trauma center. Chart reviews of included cases were conducted to gather additional details of the injury, including tenderness of the cervical spine upon palpation from the emergency department resident note, the type of C2 fracture, and the length of hospital stay. Descriptive statistics were conducted for all variables for 2 age cohorts: patients aged less than 60 years and patients aged 60 years and older. This study was reviewed and approved by our university's institutional review board.

## 3. Results

### 3.1. Demographics

**Figure 1:**
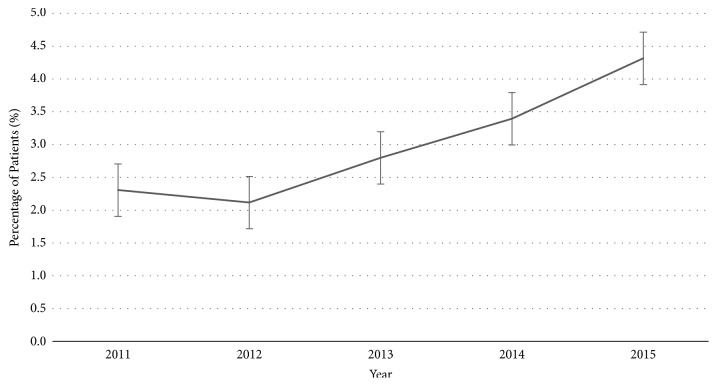
Proportion of the number of C2 fractures per study year.

**Figure 2:**
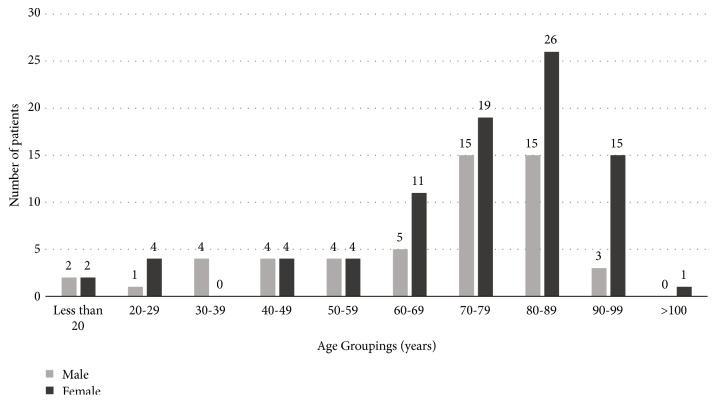
Number of patients with C2 fractures categorized by age groupings and gender.

From 2011 to 2015, a total of 18,805 patients were entered into the Trauma Registry, of which 4,621 were ≥ 60. A total of 139 patients with C2 fractures were identified during this time period (0.74%). Within each individual year of the study, the percentage of patients with C2 fractures presenting to the ED increased (Figure 1). The average age of patient presentation was 72 years, ranging from 16 to 100 years. In the overall study population, most patients were 60 years or older (79%) and female (62%). In the subgroup of patients under 60 years of age, 52% were male; however, females made up the majority of patients over the age of 60 at 65% (Figure 2).

### 3.2. Clinical Features

**Figure 3:**
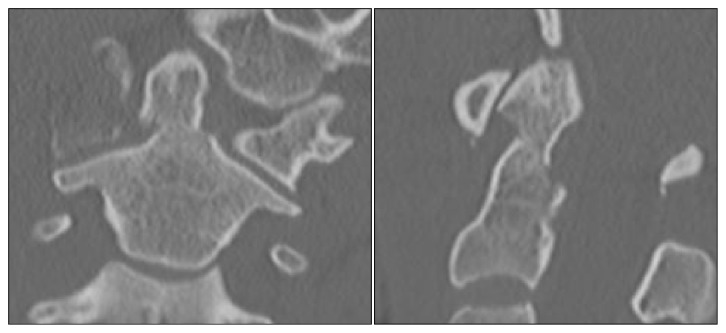
CT images in coronal (left panel) and sagittal (right panel) reconstructions, showing a 4mm displaced type II odontoid fracture.

**Figure 4:**
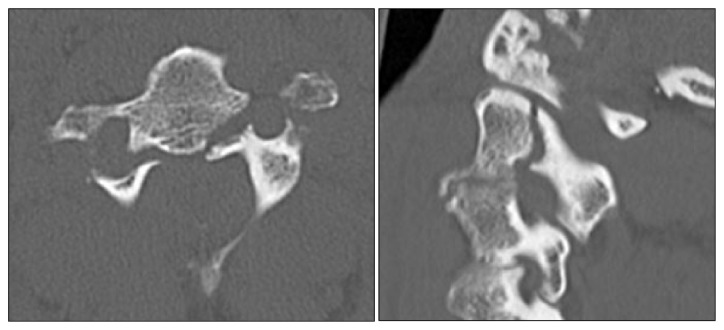
Axial (left panel) and sagittal (right panel) reconstructions showing bilateral fractures through the pars interarticularis involving the transverse foramina, consistent with a hangman's fracture.

Falls were the number one mechanism of injury among the older cohort (81%), while MVCs were more common among those under 60 (70%; Table 1). Odontoid fractures made up 85% of the C2 cervical spine fractures recorded overall, 2% were classified as hangman's fractures (*a fracture* involving the pars interarticularis of C2 on both sides), 7% were complex involving multiple aspects of C2, and 6% were other aspects of C2. Odontoid fractures accounted for the majority of the C2 fractures among patients both under and over 60 years of age (Table 2). Figures 3 and 4 provide CT images of a typical type II odontoid fracture and a hangman's fracture, respectively.

### 3.3. Reported Midline Spine Tenderness

Of the 139 patients with a C2 fracture, 116 had a documented physical exam of the cervical spine (83%). No midline spine tenderness was noted in 44 of these patients (38%) in the emergency medicine resident note in the medical record. Of the 139 patients that were part of this study, only 10 patients (7%) had C2 fractures with spinal cord involvement. Further, the majority of patients with spinal cord involvement were over 60 years of age (80%).

### 3.4. Associated Injuries

There were a total of 696 associated injuries documented across the 139 patient encounters. Isolated C2 fractures were found in 31% of patients. Skin and soft tissue injuries were the most commonly associated injury (31%). Other associated injuries are described in Table 3.

### 3.5. Other Cervical Fractures

In terms of additional spinal fractures, 32% of patients with a C2 fracture had an additional cervical spine fracture. The most commonly associated cervical spine fracture was a C1 fracture (21%) and 6% had cervical fractures at multiple levels. Of those 6%, more than half (5/9) included the C1 level as well. Finally, 17% of patients had noncervical spinal fractures, 48% had at least one additional thoracic level spinal fracture, 39% had one or more associated lumbar spinal fractures, and 9% had a combination of thoracic and lumbar spinal fractures.

### 3.6. Operative Intervention

At total of 41 patients (30%) underwent spinal intervention for their C2 fracture, whether it was the placement of a halo device or surgical intervention.  Table 4 further details what specific interventions were performed. In our study population, the majority of patients who underwent spinal intervention were over the age of 60 (n=27; 66%). In the over 60 years of age cohort, posterior fusion surgery was the most commonly performed intervention (48%). While halo devices were placed in only 8% of the over 60 age group, they were placed in 36% of the under 60 age group. Of those who underwent intervention, 17% had an associated spinal cord injury (n=7).

### 3.7. Disposition

All but one patient were admitted, with most patients being admitted to either the step-down unit (43%) or the intensive-care unit (ICU; 40%). Other dispositions included a floor unit (11%), home, observation, or the operating room (OR; 6%). The average length of stay was 6 days, with a median of 4 days. For patients who underwent spinal intervention, the average length of stay was 6.8 days with a median of 6 days. Following admission, 70 patients (50%) were discharged to home, 19% were discharged to a skilled nursing unit (SNU), and 14% were discharged to a rehab facility. Additional dispositions included acute care, leaving against medical advice, and jail (12%).

A total of 11 patients died, giving a mortality rate of 8%. Of these patients, 9 were over the age of 60. Among the patients who also had a spinal cord injury, there was a 40% mortality rate, all of which was in the over 60 age group. Of patients who underwent spinal intervention, there were 2 deaths, both of which were over 60 years of age.

## 4. Discussion

Our study demonstrated that C2 fractures are more commonly seen in older adults, with fall being the most common mechanism of injury. Cervical spine fractures have increased since 2005, with the average age of cervical spine trauma victims also rising [3, 10]. One U.S. study found that the incidence of C2 fractures increased 3-fold over a ten-year period in people over 84 years of age, while a Swedish study also found that the incidence of odontoid fractures tripled from 2000 to 2014 in those over 70 [1, 4]. This increased incidence of C2 fractures has risen at a rate even greater than the increased growth rate of the older population [11]. Thus, this injury pattern is likely to be seen with increasing frequency, as the older population is expected to double by 2050 [12].

Fall injuries can result in significant morbidity and mortality among older adults [13]. According to the Centers for Disease Control, an older person dies every 20 minutes from a fall in the United States [14]. In this study, only 50% of patients were able to return home following hospitalization, with one-third of patients requiring admission to either a rehabilitation facility or skilled nursing unit.

Odontoid fractures were the most commonly seen C2 fracture type in our older adult population at 86%. This was higher than the rates found in other studies examining C2 fracture types in older populations [4, 15]. The management of C2 fractures in older people is controversial, with some favoring surgical management and others recommending immobilization [16]. Our study reported surgical management in one-third of patients, which is similar to the 26% surgical rate found in a Swedish study examining C2 fractures [17]. Surgical management of these injuries has been increasing over time, even among older patients [4, 18]. Prior studies of older patients had mortality rates ranging from 3 to 7% [19–21]. The mortality rate of our study was slightly higher at 8%, but some patients had coexisting injuries which may have contributed to their death.

On physical examination, 62% of the patients in this study were found to have tenderness to palpation over the cervical spine. The National Emergency X-Radiography Utilization Study (NEXUS) evaluated 34,069 patients who presented with blunt trauma to 21 centers across the country and underwent cervical spine imaging to establish criteria in order to determine which patients did not require cervical spine imaging due to low probability of injury [22]. The established criteria of (1) no midline tenderness, (2) no focal neurologic deficit, (3) normal alertness, (4) no intoxication, and (5) no painful distracting injury are now used frequently in trauma centers to determine the need for imaging of the cervical spine. Although it is possible that the patients in our study had other characteristics that made their physical exam less reliable, such as dementia or distracting injuries, it is still a concern that 22 patients had no documentation of midline cervical spine tenderness. However, as this study was done in a retrospective fashion, it is possible that those patients would have been imaged according to NEXUS based upon other criteria. Nevertheless, some studies have found concerns regarding the NEXUS criteria in older populations and recommend a low threshold to image the cervical spine in these patients [23, 24]. In some instances, the Canadian C-spine rule may prove to be more useful, as an age over 65 years is considered high-risk, and imaging is suggested [25].

### 4.1. Limitations

There were several limitations to this study. First, this was a retrospective database study, which may have limited the number of analyzable variables and relies upon abstractors for completeness. Furthermore, data was collected from a single-site, meaning results may not be generalizable to other settings. Additionally, entries into the database are dependent upon the accuracy of physician documentation. For example, only 83% of patients had a documented cervical spine exam. It is possible that the exam was omitted in patients who already had a cervical spine fracture diagnosed on imaging prior to transfer to our trauma center. Only patients who presented to the hospital were included in this study; therefore, patients who died at the scene were not included. Lastly, our study did not follow longer term outcomes of these patients after hospital discharge and we were not able to determine the specific reason why some patients were discharged to long term care facility rather than returning to their residence.

## 5. Conclusions

C2 fractures are more common in older adults and usually result from falls. Most C2 fractures did not result in neurologic injury and were treated nonsurgically. A larger, prospective study should be conducted specifically on older trauma patients in order to further explore the finding of C2 fractures in the absence of midline cervical spine tenderness. A high level of suspicion for this injury should be maintained when evaluating older patients after falls, as patients may not have neck tenderness.

## Figures and Tables

**Table 1:** Mechanism of injury in C2 fracture patients with distinction between patients aged less than 60 years and those aged 60 years and older.

Mechanism of Injury	< 60 (%)	≥ 60 (%)	TOTAL (%)
Fall	7 (24)	88 (81)	95 (68)

Motor Vehicle Crash (MVC)	21 (70)	17 (15)	38 (27)

Blow to Head	1 (3)	2 (2)	3 (2)

Motorcycle Crash (MCC)	1 (3)	0 (0)	1 (1)

Altercation	0 (0)	1 (1)	1 (1)

All-Terrain Vehicle (ATV) Accident	0 (0)	1 (1)	1 (1)

*TOTAL (*%)	*30 (100)*	*109 (100)*	*139 (100)*

**Table 2:** Type of fractures.

Type	< 60 (%)	≥ 60 (%)	Total (%)
Odontoid I	1 (3)	3 (3)	4 (3)

Odontoid II	12 (41)	64 (58)	76 (55)

Odontoid III	8 (28)	28 (25)	36 (26)

Odontoid unspecified	0 (0)	1 (1)	1 (1)

Hangman's	2 (7)	1 (1)	3 (2)

Vertebral body	0 (0)	2 (2)	2 (1)

Complex	2 (7)	8 (7)	10 (7)

Other	4 (14)	3 (3)	7 (5)

*TOTAL (*%)	29 (100)	110 (100)	139 (100)

**Table 3:** Categories of associated injuries.

Type	Count (%)
Isolated C2 fracture	57 (31)

Chest/Abdomen injuries	34 (19)

Orthopedic injuries	25 (14)

Other Spine fracture	22 (12)

Facial fracture	14 (8)

Intracranial bleed	13 (7)

Vertebral artery injuries	10 (5)

Skull fracture	8 (4)

*TOTAL*	*183 (100)*

**Table 4:** Surgical interventions.

Type	< 60 (%)	≥ 60 (%)	Total (%)
Posterior Fusion	3 (21)	12 (48)	16 (39)

Anterior Fusion	3 (21)	3 (11)	6 (15)

Atlas-Axis Fusion	1 (7)	5 (19)	6 (15)

Halo Device	4 (29)	1 (4)	4 (10)

Surgical Repair unspecified	1 (7)	3 (11)	4 (10)

Halo + Fusion	1 (7)	1 (4)	3 (7)

Fusion unspecified	1 (7)	1 (4)	2 (5)

*TOTAL (*%)	*14 (100)*	*27 (100)*	*41 (100)*

## Data Availability

The authors present the data that was authorized by the institutional review board of their university. Any other additional data points from the university's Trauma Registry that was utilized would need review and approval at the request of the university's IRB.
